# Quantitative Detection of Viable but Nonculturable *Cronobacter sakazakii* Using Photosensitive Nucleic Acid Dye PMA Combined with Isothermal Amplification LAMP in Raw Milk

**DOI:** 10.3390/foods11172653

**Published:** 2022-09-01

**Authors:** Lianxia Hu, Shufei Zhang, Yuling Xue, Yaoguang Zhang, Wei Zhang, Shijie Wang

**Affiliations:** 1 College of Chemical Engineering, Shijiazhuang University, Shijiazhuang 050035, China; 2 College of Food Science and Biology, Hebei University of Science and Technology, Shijiazhuang 050018, China; 3 Junlebao Dairy Group Co., Ltd., Shijiazhuang 050221, China; 4 College of Life Sciences, Agricultural University of Hebei, Baoding 071001, China

**Keywords:** viable but nonculturable, *Cronobacter sakazakii*, propidium bromide, quantitative loop-mediated isothermal amplification, raw milk, pasteurization

## Abstract

An accurate method that rapidly detects the number of viable but nonculturable (VBNC) *Cronobacter sakazakii* was developed by combining propidium bromide with quantitative LAMP (PMA-QLAMP). The *gyrB* gene was the target for primers design. The optimal PMA treatment conditions were determined to eliminate the DNA amplification of 10^8^ CFU/mL of dead *C. sakazakii* without affecting any viable *C. sakazakii* DNA amplification. Compared with the DNA of 24 strains of common non-*C. sakazakii* strains found in raw milk and dairy products, the DNA of only six *C. sakazakii* strains from different sources was amplified using PMA-QLAMP. The ability of PMA-QLAMP to quantitatively detect non-dead *C. sakazakii* in a 10% powdered infant formula (PIF) solution was limited to 4.3 × 10^2^ CFU/mL and above concentrations. Pasteurizing 10^6^ CFU/mL viable *C. sakazakii* yielded the maximum ratio of the VBNC *C. sakazakii*. PMA-QLAMP-based detection indicated that, although approximately 13% of 60 samples were positive for viable *C. sakazakii,* the *C. sakazakii* titers in these positive samples were low, and none entered the VBNC state under pasteurization. PMA-QLAMP showed potential as a specific and reliable method for detecting VBNC-*C. sakazakii* in pasteurized raw milk, thereby providing an early warning system that indicates potential contamination of PIF.

## 1. Introduction

*Cronobacter sakazakii* belongs to the genus *Cronobacter* in the Enterobacteriaceae family [1]. As a foodborne pathogen, it is a facultative anaerobic spore-free gram-negative bacterium [2]. In 2008, according to the reports provided by the Food and Agriculture Organization and the World Health Organization, more than 120 cases of *C. sakazakii* related diseases can be seen, most of which are life-threatening infections. Many of these outbreaks were related to the consumption of infant formula contaminated with *C. sakazakii*, leading to a large number of recalls and lawsuits. In 2011, the Centers for Disease Control and Prevention reported that the number of infants infected with *C. sakazakii* had tripled and was continuing to increase [3]. *C. sakazakii* is an important foodborne opportunistic pathogen, which usually leads to diseases in newborns and people with low immunity, such as septicemia, neonatal meningitis, and necrotizing enterocolitis. Although the probability of an infant being infected with *C. sakazakii* is very low, once infected, the death rate is as high as 40–80% [4]. Most cases of *C. sakazakii* infection can be traced back to powdered infant formula (PIF), which often acts as the main source of infection [5]. Pasteurization, oligonutrition, low temperatures, or drying during the production and processing of PIF may induce *C. sakazakii* to enter a sublethal state, wherein it remains viable but cannot be cultured on a plate (VBNC) [6,7,8].

Although VBNC bacteria lose their ability to grow and reproduce on plates, their vital signs, virulence, and pathogenicity are retained. Under appropriate growth conditions, VBNC bacteria can recover, resume growth, reproduce, and quickly reach pathogenic levels [9,10]. *C. sakazakii* that has lost culturability may yet recover after periods as long as 2.5 years and cause disease following ingestion. This is compounded by the fact that such bacterial states cannot be detected via traditional plate counting methods, resulting in missed detections as well as false negatives during food safety testing [11,12]. The resulting safety risks are not easy to determine; consequently, even PIFs manufactured according to production specifications may be exposed to pollutants. Since VBNC *C. sakazakii* can be missed by plate cultures, it has been considered a “hidden pollution source”, seriously threatening the security of PIF [13,14].

Accurate identification and detection of the VBNC state have always been a major research goal in quantitatively detecting viable bacteria. Researchers have combined methods based on different principles to more rapidly and effectively detect the number of viable bacteria [15,16]. Currently, approaches combining viable bacteria counting methods and culturable bacteria counting methods are widely used. Therein, the quantitative value of VBNC bacteria is calculated as the difference between the significantly higher value of viable bacteria and the plate culture value. Generally, an accurate estimate of culturable bacteria can be obtained via the plate counting method. Therefore, difficulties associated with VBNC bacterial detection are mostly linked to viable bacteria counting methods [17,18]. Using the PMA-qPCR technology of Cao et al. [19], the 9.08 Log10 CFU/mL concentration of the viable but nonculturable (VBNC) state of *C. sakazakii* in the later stage of milk fermentation could be detected. Lv et al. [20] developed the IMS-PMAxx-ddPCR method after IMBs enrichment, which showed higher detection of *C. sakazakii* VBNC in PIF (lower limit was 5.6 copies/g).

Quantitative isothermal amplification (QLAMP) conducts a molecular biological method to detect nucleic acids [21], where fluorescent dyes are added to a conventional LAMP (loop-mediated isothermal reaction) reagent tube [22]. Based on the positive proportional relationship between the fluorescent intensity of dye and the DNA concentration (double-stranded), the concentration of initial DNA can be quantified, a technique that shows advantages, such as strong specificity, high sensitivity, rapidity, and accuracy. However, the nucleic acid amplification method does not distinguish between dead and viable bacteria in the sample. After bacteria die, the DNA remains long-term on food, which may lead to false positives, overestimates of the actual bacterial abundance, and inaccurate test results. Propidium monoazide (PMA) combined with nucleic acid amplification is currently used to quantitatively detect viable bacteria [23]. PMA is a selective nucleic acid crosslinking dye, which can penetrate the membranes of dead bacteria but not those of viable bacterial cells [24,25]. After entering the cell, PMA covalently cross-links with DNA after more than 1 min of intense light exposure. At the same time, free PMA outside the cells is removed via photolysis to avoid false-negative results. Thus, target DNA in dead cells will not be amplified [26,27]. The bacteria in the VBNC state are viable bacteria without cell membrane damage. The combination of PMA and QLAMP Technology (PMA-QLAMP) can quantitatively measure viable bacteria, including the proportions of VBNC bacteria.

In this study, primers were designed based on *gyrB* [28] of the *C. sakazakii* species-specific gene, while the PMA-QLAMP method was established to quickly and accurately detect viable *C. sakazakii*. Some *C. sakazakii* can be induced into the VBNC state by pasteurization. Selective inhibition of amplification of nonviable *C. sakazakii* DNA by PMA in PIF, thereby providing a reference for the monitoring and inspection of foodborne pathogenic bacteria in the VBNC state.

## 2. Materials and Methods

### 2.1. Strains and Culture Conditions

The specificity of this set of primers was verified by detecting six *C. sakazakii* (ATCC29544, CICC21561, CICC21562, CICC21572, CICC24112, CICC21665) strains and 24 related bacterial strains by the QLAMP. Single colonies from each of the six *C. sakazakii* strains were cultured for 18 h at 37 °C in a buffer peptone water (BPW) culture medium. Single colonies from each of the eight *Pseudomonas* strains, (*Pseudomonas fluorescens* ATCC13525, *Pseudomonas aeruginosa* CICC10351, *Pseudomonas alcaligenes* CICC23927, *Pseudomonas simiae* CICC22692 CICC10402, *Pseudomonas chlororaphis* CICC21627, *Pseudomonas rhodesiae* CICC21957, *Pseudomonas putida* CICC21624) were inoculated into BHI (i.e., brain heart infusion) broth and cultured at 30 °C for 18 h. *Streptococcus thermophilus* CICC20174, *Lacticaseibacillus paracasei* CGMCC4691, and five strains *of Lactobacillus* (CGMCC1.3342, CGMCC1.1856, CGMCC1.120, CGMCC1.2442, and CGMCC1.580) were inoculated in MRS (de Man, Rogosa, and Sharpe) agar free medium, respectively, and cultured at 37 °C for 24 h. Single colonies of the nine related bacterial strains (*Escherichia coli* CMCC44102, *Salmonella* Typhimurium CICC21484, *Staphylococcus aureus* ATCC25923, *Listeria monocytogenes* ATCC19115, *Bacillus cereus* CICC20450, *Bacillus licheniformis* ATCC21424, *Streptococcus agalactiae* ATCC 13813, *Streptococcus mutans* ATCC35668, *Streptococcus gordonii* ATCC 49818) were incubated in NB (nutrient broth) medium under their respective suitable culture conditions for 18 h. Bacterial strains were purchased from the ATCC (American Type Culture Collection), the CGMCC (China General Microbiological Culture Collection), the CMCC (National Center for Medical Culture Collection), and the CICC (China Industrial Culture Collection). The Co., Ltd. of China Beijing land bridge technology provided various culture media for this study.

### 2.2. Preparation of Bacterial DNA

The precipitates of 1 mL of suspensions containing exponentially growing bacteria were collected by centrifugation (13,523× *g*, 2 min, 20 °C). Next, 130 μL of sterile water and 70 μL lysozyme (50 mg/mL) were added to a centrifuge tube containing a solution of gram-positive bacteria, which was vibration-mixed and allowed to stand at 20 °C for 5 min. After centrifugation (13,523× *g*, 2 min, at 20 °C) to collect the precipitates, all strains were subjected to DNA extraction according to the lysis of boiling water method described by Wang et al. [29]. The extracted DNA of all strains was placed at −20 °C for subsequent experiments.

### 2.3. Construction of PMA Treatment Method

#### 2.3.1. Selection Time of Heat Killing for *C. sakazakii*

Each 1 mL of *C. sakazakii* viable bacteria suspension (10^8^ CFU/mL) was filled in a centrifuge tube and heated for 0, 1, 2, 4, 6, 7, 8, 9, or 10 min at 100 °C. After the heating period, each tube was immediately cooled by placing it on ice. Thermal lethal time in the heat-treated bacterial suspension was determined as a Drugan-Forsythe-Iversen agar (DFI, Oxoid Thermo Fisher, Altrincham, United Kingdom) plate counting [30,31] of <1 CFU/mL (three replicates in each group) and a negative PMA-QLAMP detection (not PMA-treated bacteria were used as the control, three replicates in each group). DFI plate counting was used as a method for gradient dilution of *C. sakazakii* (according to the requirements of GB 4789.2-2016) and culture counting. *C. sakazakii* (1 mL) was added to each of two sterile culture dishes, and 20 mL of DFI medium (cooled to 46 °C) was injected into each culture dish and rotated to mix evenly. After the agar was coagulated, the plate was turned over and incubated at 36 °C for 24 h before counting.

#### 2.3.2. Establishment of PMA Pretreatment System for *C. sakazakii*

The orthogonal test (three factors: PMA working concentration (A), the time of dark reaction (B), and exposure (C); three contents (levels): (1) 3 μg/mL (working concentration), 3 min (avoid light), 3 min (exposure); (2) 5 μg/mL (working concentration), 5 min (avoid light), 5 min; (3) 10 μg/mL (working concentration), 10 min (avoid light), 10 min (exposure) of PMA (40013, Biotium, Hayward, CA, USA) was designed as shown in Table 1. The best PMA pretreatment system was based on the analysis and selection of nine groups of experimental results, as described by Van et al. [32], consisting of a combination of three factors that can completely inhibit the dead *C. sakazakii* DNA amplification while exerting no effect on the viable *C. sakazakii* DNA amplification.

Next, 0.3, 0.5, and 1 μL of a 10 mg/mL PMA solution were added to *C. sakazakii* suspension (1 mL) solutions to form three levels of PMA working concentration, respectively, which were protected from light for 3, 5, and 10 min, respectively. Each mixture treated with the PMA of *C. sakazakii* (placed on ice) was exposed under a halogen light source (650 W, Philips, Italy) placed 20 cm above the specimen for 3, 5, and 10 min, respectively. The sediment was then collected by centrifugation (13,523× *g*, 2 min, 20 °C), resuspended twice with sterile water (200 µL), and DNA (lysed in the 100 µL sterile water) was extracted according to Section 2.2 Preparation of bacterial DNA. QLAMP was used to detect the PMA-treated and -untreated DNA to analyze the inhibition rate (maximum) of dead bacteria DNA. The inhibition rate (minimum) of viable bacteria was analyzed by DFI agar plate counting. The best conditions for PMA treatment were determined by comprehensive consideration.

### 2.4. Development of the QLAMP Method

#### 2.4.1. Screening of Species-Specific Gene Sequences and Design of QLAMP Primers

The *gyrB* gene (1156 bp) of *C. sakazakii* ATCC 29544 (GeneBank ACC No: JX983606) was obtained from GenBank and submitted to BLASTN of NCBI (National Center Biotechnology Information, available at https://blast.ncbi.nlm.nih.gov/Blast.cgi?PROGRAM=blastn&PAGE_TYPE=BlastSearch&LINK_LOC=blasthome (accessed on 10 June 2021)) to search for related sequences. Subsequently, 23 strains sequences (ten *C. sakazakii* and 13 related strains) showing more than 92% homology with *C. sakazakii* ATCC 29544 were downloaded. Using Clustalx software (version 1.81, European Bioinformatics Institute, the United Kingdom, available at https://mydown.yesky.com/pcsoft/33463932/versions/ (accessed on 5 March 2021)), 23 sequences were shortened into equal length (520 bp) sequences. Five identical sequences were deleted from these 23 with DAMBE software (version 7.0.35, Kluwer Academic, Boston, available at https://www.softpedia.com/get/Science-CAD/DAMBE.shtml (accessed on 5 March 2021)). The phylogenetic tree of the retained 18 sequences targeting *gyrB* was constructed. The robustness of each branch was evaluated with bootstrapping replicates (1000 times, neighbor-joining) using MEGA software (version 4.0, Temple University, Philadelphia, PA, USA, available at https://mydown.yesky.com/pcsoft/107253376/versions/ (accessed on 5 March 2021)). The phylogenetic tree was used to analyze whether this sequence represented the sequence of the intraspecific conserved region and interspecific variation region of *gyrB* of *C. sakazakii* ATCC 29544.

The sequences of the intraspecific conserved region and interspecific variant region of gyrB of *C. sakazakii* ATCC 29,544 were determined by aligning. Six QLAMP primers were designed on line primerexplorer (version V5, EIKEN CHEMICAL, Japan, its website was https://primerexplorer.jp/elamp5.0.0/index.html (accessed on 12 June 2021)), including two outer primers FOP (the 13 bp base sequence 5′-3′ direction was ‘CCGTTAAAGTGCC’) and BOP (the 14 bp base sequence 5′-3′ direction was ‘GCTTTACGTGCCGC’), two inner primers FIP (the 40 bp base sequence 5′-3′ direction was ‘TGCTGCTCAACCGCCGATTTCTCCTCCCAGACCAAAGACA’) and BIP (the 36 bp base sequence 5′-3′ direction was ‘GATGAACGAGCTGCTGGCCGTCGATAATTTTGCCGA’), two loop primers FLP (the 16 bp base sequence 5′-3′ direction was ‘CACCTCGGAGGAGACC’) and BLP (the 14 bp base sequence 5′-3′ direction was ‘CTGCTGGAGAACCC’).

#### 2.4.2. Quantitative LAMP Amplification Reaction

First, reaction buffer (2.5 μL, 10× Thermopol, new England biolabs (NEB), MgSO_4_, 0.5 μL, 100 mM, NEB, Ipswich, MA, USA), dNTPs (1.5 μL, an equimolar mixture of dATP, dCTP, dGTP, and dTTP each at 2.5 μmoL, TaKaRa Bio (Dalian) Co., Ltd., Dalian, China), betaine (2.0 μL 10 M, Sigma, Ronkonkoma, NY, USA), primer (2.5 μL each of FIP, BIP, FLP, and BLP, 0.5 μL each of FOP and BOP, 10 μM, Shanghai Bio technology Co., Ltd., Shanghai, China), SYBR Green I (10,000×, 1:200, 0.3 μL, Coolaber Science and Technology Co., Ltd., Beijing, China), DNA template (1 μL, 10 ng/μL < initial concentration < 100 ng/μL), BST DNA polymerase large fragment (1.0 μL, 8 U/μL, NEB, Ipswich, MA, USA) were mixed with 4.7 μL of water (sterile distilled, TaKaRa Bio (Dalian) Co., Ltd., Dalian, China) in a PCR tube (200 μL, Axygen Scientific Inc., Union City, CA, USA) up to a total volume to 25 μL. The reaction system was mixed evenly and briefly centrifuged. Then the liquid surface of the amplification system was covered with 20 μL of mineral oil to prevent the volatilization of amplification products from polluting the amplification area. Last, the reaction PCR tube was placed in an amplifier (Applied Biosystems Quantum Studio 3 System, Waltham, MA, USA). A constant temperature was set at 62 °C for 40 min, generating a real-time amplification curve.

#### 2.4.3. Specificity of QLAMP

The DNA of six *C. sakazakii* strains from different sources and 24 related bacterial strains from milk and dairy products were detected using QLAMP to verify the primer specificity. The DNA contents of the 1 mL bacterial suspensions that had reached the exponential growth stage in 30 *C. sakazakii* and non-C. *sakazakii* strains ranged from 45–55 ng/μL. The DNA of blank control was replaced by sterile distilled water. The DNA was extracted (boiling water lysis method) and detected twice.

#### 2.4.4. Detection Limit and Quantitative Range of Viable *C. sakazakii*

*C. sakazakii* ATCC 29544 liquid culture suspension (1 mL, cultured for 18 h at 37 °C) was counted using DFI agar plate count. Meanwhile, after PMA treatment, 1 mL of continuously diluted (tenfold) solution of *C. sakazakii* ATCC 29544 was centrifuged (13,523× *g*, 2 min) and a series of concentrations of DNA (100 μL each) were extracted according to the boiling water bath method in Section 2.2 “Preparation of bacterial DNA”. Each series of DNA solutions was detected three times with PMA-QLAMP. The logarithm of the initial concentration of viable *C. sakazakii* was set as the abscissa, and the Ct value (1 cycle (average Ct value of three measurements) = 1 min) was set as the ordinate, and the standard curve was drawn. When quantifying *C. sakazakii*, the initial concentration of *C. sakazakii* was calculated according to the logarithm of the concentration corresponding to the Ct value on the standard curve when the peak of *C. sakazakii* starts. On the standard curve, the amplification efficiency E [33] was also calculated by the slope S of the linear equation using the following formula:

E = 10^−1/s^ − 1,

We ensured that there was no *C. sakazakii* in the PIF via DFI agar plate counting and PCR methods [34]. The experiments for the detection limit of *C. sakazakii* in 10% PIF solution by PMA-QLAMP were as follows: First, a 10% PIF solution was prepared by adding 25 g of PIF to 225 mL of water at 50–60 °C in a beaker. This was stirred to dissolve the PIF, then autoclaved at 115 °C for 15 min. Second, the DFI agar plate counting method and PCR amplification sequencing method [34] were used to confirm there was no *C. sakazakii* in the 10% PIF solution. For DFI agar plate counting, 1 mL of 10% PIF solution was used. At the same time, a parallel test was conducted using 1 mL petroleum ether, 1 mL absolute ethanol, and 1 mL ammonia added to 1 mL of 10% PIF solution, which were mixed by vibration to resuspend the precipitate. DNA was extracted according to Section 2.2 “Preparation of bacterial DNA”. The universal primers 341F (5′-CCTACGGGNGGCWGCAG-3′) and 806R (5′-GGACTACHVGGGTATCTAAT-3′) of bacterial 16S rRNA sequence were used to amplify the DNA according to the PCR amplification reaction system and the conditions of Guo et al. [34]. The PCR amplification products were sequenced, and the results were aligned with the *C. sakazakii* sequence from GenBank. Comparison of the PCR amplification products sequences showed no homology with *C. sakazakii* sequences, confirming there was no growth of *C. sakazakii* colonies on the DFI agar plate and no *C. sakazakii* in the 10% PIF solution, allowing it to be used as a sample for artificial quantitative addition of *C. sakazakii*.

The specific operation of the artificial quantitative addition of *C. sakazakii* was as follows: Firstly, *C.* *sakazakii* BPW suspension was concentrated. 10 mL of *C. sakazakii* BPW suspension (incubated at 37 °C for 18 h) were centrifuged (13,523× *g*, 2 min); 1 mL of sterile water was added to the tube and vibration-mixed into a concentrated suspension of the initial *C. sakazakii*, which was added to 9 mL of 10% PIF solution. Next, a 1 mL viable *C. sakazakii* suspension from the 10 mL of 10% PIF solution was sampled and serially diluted (ten times) before plate counting. Next, 1 mL serially diluted solutions were placed in 5.0 mL sterile centrifuge tubes, and 1 mL petroleum ether, 1 mL absolute ethanol, and 1 mL ammonia were added to the solution, which was vibration-mixed to resuspend the sediment. The obtained sample suspensions were centrifuged (13,523× *g*, 2 min). After discarding the supernatant, 1 mL of sterile water was added to each centrifuge tube, which was vibrated to resuspend the sediment. Finally, serially diluted suspensions of viable *C. sakazakii* ATCC 29544 were treated with PMA, centrifuged, and collected. A series of DNA lysates (100 μL, boiling water bath method) were detected in triplicates with QLAMP. The average Ct value was calculated by averaging three Ct values to determine the PMA-QLAMP detection limit using artificially added viable *C. sakazakii* ATCC 29544 to PIF.

### 2.5. Pasteurization-Induced Preparation of the VBNC State of C. sakazakii

*C. sakazakii* strain was activated once in BPW medium at 37 °C for 18 h, and subcultured twice under the same conditions. After being activated once and subcultured twice, *C. sakazakii* was cultured for 18 h at 37 °C in BPW culture medium. At this time, the bacteria is in an exponential growth period. It can be considered that the bacteria in this period are almost all viable bacteria. The viable *C. sakazakii* concentration can be accurately obtained by DFI agar plate counting. Seven centrifuge tubes containing 10% PIF were prepared. Subsequently, 1 mL viable *C. sakazakii* suspension was added into each tube containing 10% PIF to generate serially diluted (10^7^ CFU/mL to 10^1^ CFU/mL) concentrations. A total of nine replicates were obtained for each heated group (65 °C, 30 min), with non-heat-treated viable *C. sakazakii* suspension as the control group (three replicates in each group). Next, the centrifuge tubes were placed on ice for immediate cooling, following which the viable *C. sakazakii* concentration in each heat-treated and non-heat-treated suspension was measured via DFI agar plate counting (three replicates in each group). At the same time, the bacterial suspensions were treated with PMA (three replicates in each group) and without PMA as the control group (three replicates in each group). DNA was extracted from bacterial suspension (boiling water bath method), and the upper lysate was used as a template for QLAMP detection and analysis.

### 2.6. Changes in the Number of Viable C. sakazakii in Raw Milk Samples before and after Pasteurization

The numbers of viable *C. sakazakii* in 60 raw milk samples (these samples were pasteurized, while non-sterilized raw milk samples were used as a control) obtained from individual dairy farmers in Hebei Province, China, were estimated using DFI agar plate counting and PMA-QLAMP methods. With respect to the plate counting method, 1 mL of sample was evenly mixed with approximately 20 mL of sterilized solid DFI agar medium, which was unsolidified (water bath was maintained at 45 °C) and cultured at 36 °C for 24 h after the medium was solidified (three parallel tests were conducted at the same time). Next, 1 mL each of the raw milk sample before and after pasteurization was collected in two 5.0 mL sterile centrifuge tubes, respectively, following which 1 mL anhydrous ethanol, 1 mL ammonia, and 1 mL petroleum ether were added into each 5.0 mL sterile centrifuge tube. Each centrifuge tube was vibrated to resuspend the sediment, and the obtained suspension was centrifuged (13,523× *g*, 2 min). The supernatant was discarded, and 1 mL of sterile water was added. Each centrifuge tube was vibrated to resuspend the sediment and treated with PMA. The DNA was extracted (boiling water method) three times from each sample, and QLAMP was detected in two of the three DNA samples. Plate counting and PMA-QLAMP methods were used to compare and analyze the count changes of viable *C. sakazakii* in raw milk samples before and after pasteurization.

### 2.7. Statistical Analysis

SPSS 17.0 was used to carry out the three factor and three level design of the orthogonal experiment, and Excel 2007 was used to calculate the average value to analyze the results of the orthogonal experiment. Excel 2007 was used to calculate the mean value and standard deviation of the quantitative detection results of *C. sakazakii* VBNC state, and the data results were shown as mean ± standard deviation. All experiments were repeated three times.

## 3. Results

### 3.1. Determination of PMA Optimal Treatment Conditions

After heating each 1 mL suspension of *C. sakazakii* in a water bath at 100 °C for 0, 1, 2, 4, 6, 7, 8, and 9 min, respectively, plate counting revealed that the concentration of viable *C. sakazakii* was 4.5 × 10^8^, 2.3 × 10^6^, 1.2 × 10^6^, 4.8 × 10^3^, 1.7 × 10^2^, 6.0 × 10^0^, <1, and <1 CFU/mL, respectively. After heat treatment of 10^8^ CFU/mL *C. sakazakii* suspension for 8 min, no colonies grew on the plate, indicating sterilization. Therefore, 8 min was the heat death time of the 10^8^ CFU/mL *C. sakazakii* suspension.

K1 was the average value of the inhibition rate under PMA treatment conditions of level 1 (PMA working concentration was 3 μg/mL, dark reaction time was 3 min, exposure time was 3 min). K2 was the average value of the inhibition rate under PMA treatment conditions of level 2 (PMA working concentration was 5 μg/mL, dark reaction time was 5 min, exposure time was 5 min). K3 was the average value of the inhibition rate under PMA treatment conditions of level 3 (PMA working concentration was 10 μg/mL, dark reaction time was 10 min, exposure time was 10 min). R was the maximum value minus the minimum value of the average inhibition rate under each PMA treatment factor (the three factors were PMA working concentration, dark reaction time, and exposure time, respectively). Table 2 shows that the inhibition rate of group 8 test results was 99.98%. This shows that it not only had the highest inhibition rate among the nine groups of test results but also had no effect on the amplification of viable *C. sakazakii* DNA. The combination of factors and levels corresponding to this result was A3B2C1. The best treatment conditions (final PMA concentration, reaction time in the dark, and exposure time) were 10 μg/mL, 5 min, and 3 min, respectively.

### 3.2. Determination of Species-Specific Gene Sequences of C. sakazakii

**Figure 1:**
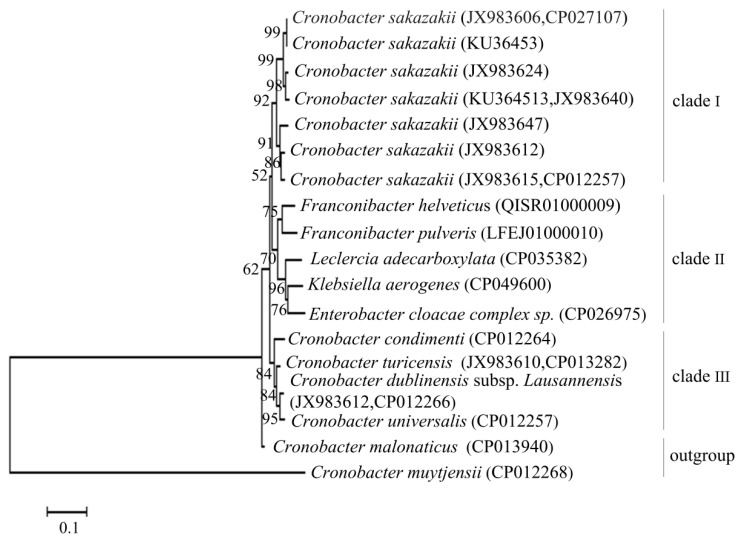
Phylogenetic tree was constructed by the *gyrB* gene of *C. sakazakii* and its related species. *C. sakazakii* was clustered on a branch (Clade I).

Figure 1 showed that the phylogenetic tree was constructed by the *gyrB* gene of *C. sakazakii* and its related species. The tree was divided into four main branches: clade I (only *C. sakazakii* one species), clade II (consisted of five species: *Franconibacter helveticus*, *Franconibacter pulveris*, *Leclercia adecarboxylata*, *Klebsiella aerogenes*, and *Enterobacter cloacae complex* sp.), clade III (consisted of four species: *Cronobacter turicensis*, *Cronobacter condimenti Cronobacter dublinensis* subsp. *Lausannensis* and *Cronobacter universalis*), and the outgroup (consisted of two species: *Cronobacter malonaticus* and *Cronobacter muytjensii*). In this case, 10 strains of *C. sakazakii* were clustered on a branch with a 92% bootstrap value, which differs from other species in this phylogenetic tree, indicating that the *gyrB* sequence of *C. sakazakii* showed high intraspecific homology and interspecific specificity, suitable for designing PMA-QLAMP primers.

### 3.3. Verification Results of PMA-QLAMP Specificity

**Figure 2:**
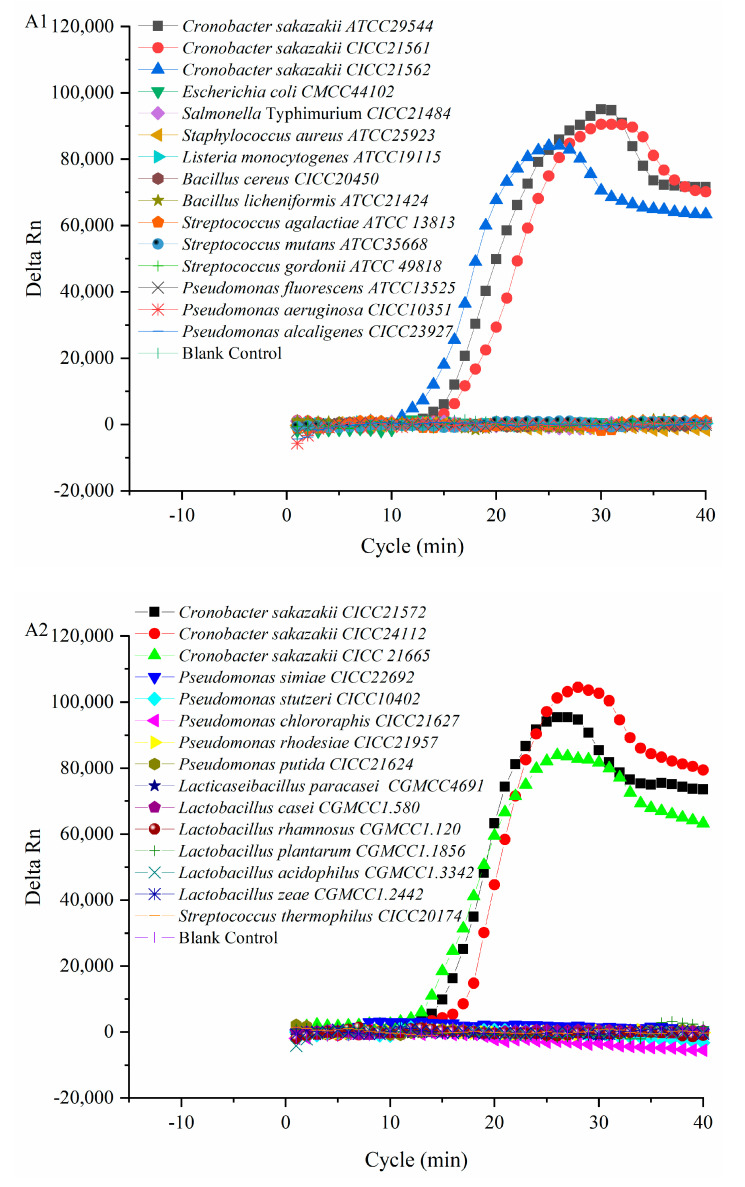

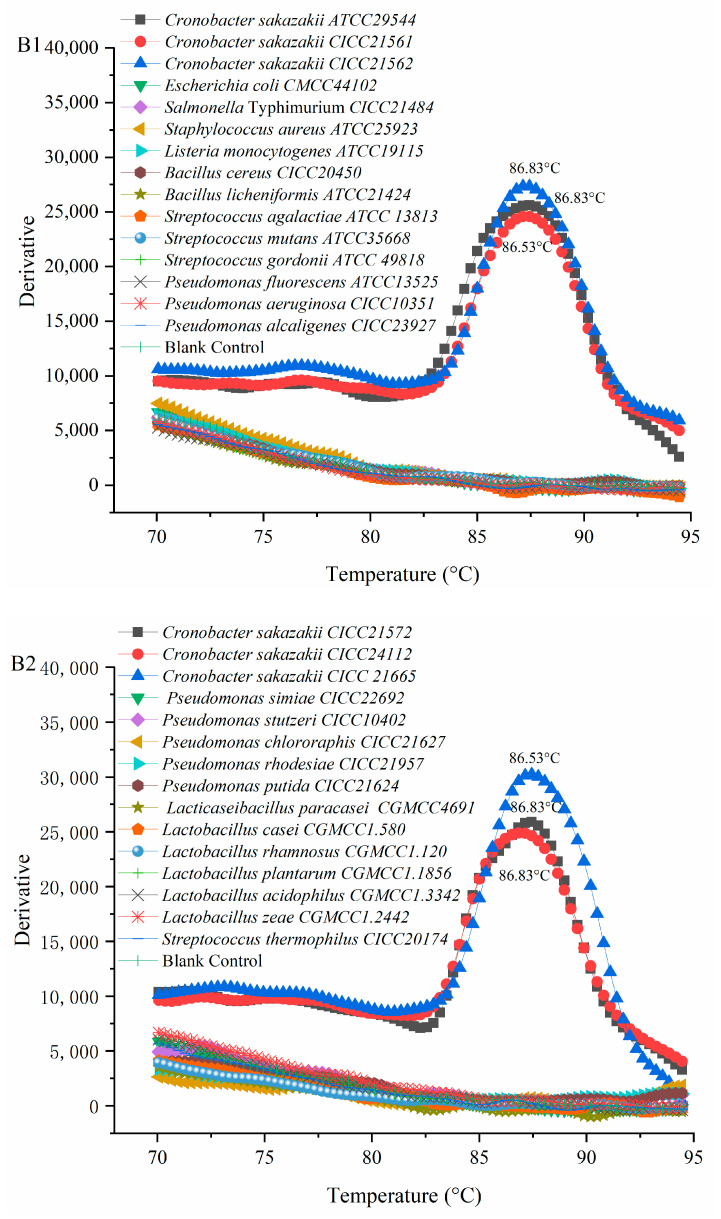
Specificity verification of PMA-QLAMP method. (**A1**,**A2**) Specific amplification curve of PMA-QLAMP. That was, the *C. sakazakii* amplification curves of ATCC29544, CICC21561, and CICC21562 in (**A1**), CICC21572, CICC24112, and CICC2166 in (**A2**) all showed S-shaped curves. The amplification curve of 24 non-*C. sakazakii* strains and the blank control showed a horizontal straight line, and the fluorescence intensity (delta Rn) remained at about 0. (**B1**,**B2**) Melting curve of the amplified products PMA-QLAMP. In the temperature range of 70–95 °C, i.e., ATCC29544, CICC21561, and CICC21562 in (**B1**), CICC21572, CICC24112, and CICC2166 in (**B2**), the melting curve of the amplified products of *C. sakazakii* was unimodal, and the X-axis temperature corresponding to the highest peak of the curve was the melting temperature. In (**B1**), the melting temperatures of *C. sakazakii* ATCC29544, CICC21561, and CICC21562 were about 86.83, 86.53, and 86.83 °C, respectively. In (**B2**), the melting temperatures of *C. sakazakii* CICC21572, CICC24112, and CICC2166 were about 86.83, 86.83, and 86.53 °C, respectively. The melting curve of 24 non-*C. sakazakii* strains and the blank control were a straight line, respectively. They had no unimodal curve and corresponding melting temperature.

Six *C. sakazakii* and 24 of non-*C. sakazakii* strains were used to verify the specificity of the PMA-QLAMP method. The fluorescence intensity of the PMA-QLAMP reaction system increases continuously (delta Rn), showing an S-shaped curve, indicating that the DNA of the *C. sakazakii* strain was amplified. The fluorescence intensity (delta Rn) of DNA and blank control of 24 non-*C. sakazakii* strains remained around 0, indicating that their DNA (or no DNA) was not amplified (Figure 2(A1,A2)). The melting temperature for *C. sakazakii* amplified products with PMA-QLAMP was almost the same, about 86.83 °C (Figure 2(B1,B2)). The lack of melting temperature amplified products of 24 non-*C. sakazakii* strains and blank control indicated that no nonspecific amplification reaction and no pollution occurred during PMA-QLAMP, respectively.

### 3.4. The Detection Limit and Quantitative Range of PMA-QLAMP for Viable C. sakazakii

**Figure 3:**
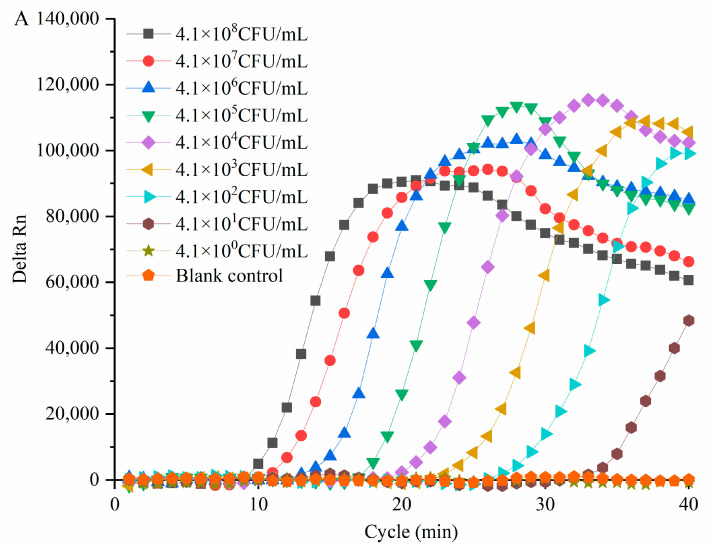

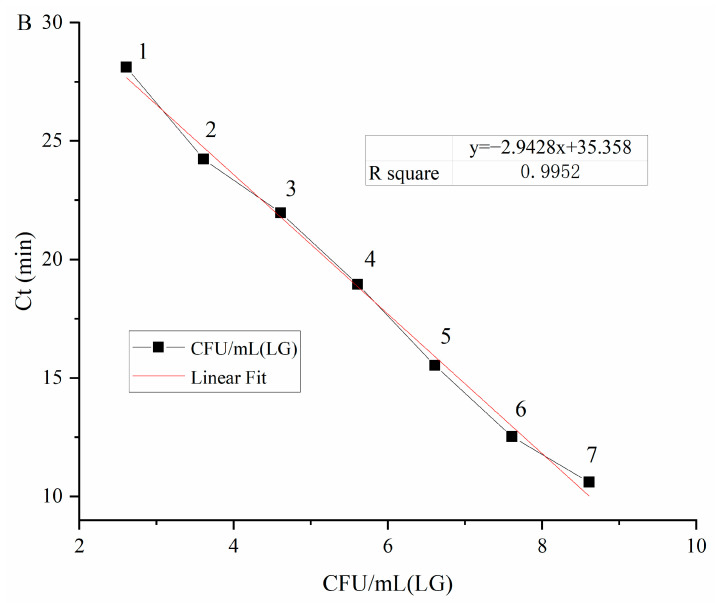
The quantitative detection limit and linear relationship of PMA-QLAMP for 10 series of dilution concentrations of viable *C. sakazakii*. (**A**) A 10-point *C. sakazakii* suspension serial dilution led to concentrations ranging from 4.1 × 10^8^ to 4.1 × 10^2^ CFU/mL, with average Ct values of 10.61, 12.52, 15.53, 18.96, 21.97, 24.24, 28.12, and 34.28 min, respectively. The interval (about 3 min) was the same and uniform between every two adjacent Ct values. For *C. sakazakii* suspension concentrations of 4.1 × 10^2^ and 4.1 ×10^1^ CFU/mL, the Ct values were 28.12 and 34.28 min, respectively. The interval (6.16 min) became significantly larger between 28.12 and 34.28 min. (**B**) Draw a standard curve. From the standard curve, there was a linear relationship (y = −2.9428x + 35.358, R^2^ = 0.9952) between Ct values (10.61–28.12 min) and log concentrations (no. 7–1: 8.61, 7.61, 6.61, 5.61, 4.61, 3.61, and 2.61, respectively) of viable *C. sakazakii*.

Viable *C. sakazakii* ATCC29544 suspensions with concentrations ranging from 4.1 × 10^8^ CFU/mL to 4.1 × 10^0^ CFU/mL (plate count) were used to determine the detection limit of PMA-QLAMP for viable *C. sakazakii* in pure culture. PMA-QLAMP detected every 10 dilutions of *C. sakazakii* DNA as a template. It can be seen from the amplification curve in Figure 3A. CT values corresponding to 10 series of dilution concentrations were obtained. The average Ct values were calculated by repeatedly measuring Ct values for each gradient three times; the Ct values of each serial dilution were 10.61, 12.52, 15.53, 18.96, 21.97, 24.24, 28.12, and 34.28 min, respectively. Took the average Ct values as the ordinate and the logarithm of *C. sakazakii* concentration (log_10_ CFU/mL, i.e., l g CFU/mL), corresponding to the DNA template as the abscissa to draw the standard curve as shown in Figure 3B. The linear equation was as follows:

y = −2.9428x + 35.358.

It can be seen from the standard curve that R^2^ = 0.9952 > 0.99 means that the equation had a good linear relationship. The logarithm of viable *C. sakazakii* concentration was in the range of 8.61–2.61, which meant that accurate quantification could be carried out. The detection limit of PMA-QLAMP for viable *C. sakazakii* was 4.1 × 10^2^ CFU/mL in pure culture, and the quantitative range was 4.1 × 10^8^–4.1 × 10^2^ CFU/mL CFU/mL.

PMA-QLAMP detection limit for viable *C. sakazakii* ATCC29544 added in the 10% PIF solution with concentrations (plate count) ranging from 4.3 × 10^8^ to 4.3 × 10^0^ CFU/mL was determined. Average Ct values of the dilutions were 9.80, 12.08, 15.77, 18.15, 22.36, 24.22, 27.69, and 34.00 min. The Ct values (13.08, 16.77, 19.15, 22.36, 24.22, and 27.69 min) were determined using the standard curve; all remained within the accurate quantitative range (10.61–28.12 min). The corresponding concentration of *C. sakazakii* at the maximum quantitative Ct value (27.69 min) was 4.3 × 10^2^ CFU/mL. PMA-QLAMP detection limit for viable *C. sakazakii* in the 10% PIF solution was 4.3 × 10^2^ CFU/mL.

### 3.5. Quantitative Detection Results of C. sakazakii VBNC State

A series of diluted viable *C. sakazakii* concentrations in 10% PIF solution were detected using QLAMP. Before pasteurization, the measured values of the serial concentrations of viable *C. sakazakii* were expressed by logarithm viable *C. sakazakii* concentrations, and the mean ± standard deviation (Table 3), which were 7.64 ± 0.02, 6.67 ± 0.04, 5.72 ± 0.01, 4.8 ± 0.03, 3.87 ± 0.02, 2.8 ± 0.02, and 1.76 ± 0.04, respectively. Following pasteurization at 65 °C for 30 min, the series of diluted viable *C. sakazakii* concentrations in 10% PIF solution were measured by plate counting. At 10^4^ CFU/mL, the logarithm of bacterial concentration was 0, the corresponding logarithm of bacterial number detected by PMA-QLAMP was 2.93, and the logarithm of bacterial number detected by PMA-QLAMP at 10^2^ CFU/mL was 0. At 10^6^ CFU/mL, the highest proportion of VBNC state of *C. sakazakii* was 15.32%. The results showed that pasteurization of 10^6^ CFU/mL of viable *C. sakazakii* resulted in the largest ratio of viable *C. sakazakii* entering the VBNC state. By contrast, pasteurization of <10^2^ CFU/mL viable *C. sakazakii* did not trigger the *C. sakazakii* VBNC state.

### 3.6. Quantitative Detection Results of Viable C. sakazakii in Raw Milk Samples

Sixty samples of bulk raw milk from individual dairy farmers in Hebei Province were evaluated using the DFI agar plate counting method. The results showed that viable *C. sakazakii* was not detected. PMA-QLAMP was used to detect viable *C. sakazakii* numbers of positive samples in bulk raw milk before pasteurization. The PMA-QLAMP detection rate of samples positive for viable *C. sakazakii* reached 13% (Table 4). Ct values ranged between 24.24 and 28.12, and PMA-QLAMP accurately and quantitatively detected that the concentrations of viable *C. sakazakii* in three raw milk samples ranged between 4.3 × 10^3^ and 4.3 × 10^2^ CFU/mL. The concentration of viable *C. sakazakii* in five raw milk samples detected by MA-QLAMP ranged between 4.3 × 10^2^ and 4.3 ×10^0^ CFU/mL, with Ct values between 28.12 and 40, indicating low *C. sakazakii* concentrations with the range of uncertainty. Following pasteurization, PMA-QLAMP did not detect viable *C. sakazakii* in the 60 raw milk samples. This indicated that low concentrations of viable *C. sakazakii* had not formed any *C. sakazakii* in the VBNC state following pasteurization.

## 4. Discussion

In this study, the optimized PMA treatment conditions (PMA working concentration of 10 μg/mL, dark reaction time of 5 min, exposure time of 3 min) inhibited the DNA amplification of 10^8^ CFU / mL dead cells of *C. sakazakii*, while the DNA amplification of viable *C. sakazakii* was not affected by QLAMP, and the amplification efficiency of QLAMP was 119%. The results were consistent with of Liu et al. [35], who found that PMA completely inhibited PCR amplification of *E. sakazakii* with a minimum PMA concentration of 5 μg/mL, and the maximum PMA concentration that did not inhibit PCR amplification of viable E. sakazakii was 15 μg/mL. Therefore, the orthogonal test with a reasonable design of key factors and levels can accurately and quickly complete the optimization of PMA treatment conditions with the least testing.

As an important molecular marker, the *gyrB* gene sequence is a conservative housekeeping gene helpful for bacterial phylogenetic analyses [36]. The single copy number of nuclear genomic DNA is small and relatively constant compared with other genes, so that it can be accurately and stably detected [37]. Ma et al. [38] constructed a plasmid standard sample with the *gyrB* gene of *C. sakazakii* as the target sequence, and the plasmid was stable after 15 passages. In this study, primers were designed according to the specific sequence of *C. sakazakii gyrB* species. The analysis of gene sequences from the biological evolutionary tree and the detection of actual strains showed that the *gyrB* gene sequence of *C. sakazakii* could be distinguished and identified at the species level. Chen et al. [39] sequenced the *gyrB* gene sequence (1066 bp) of *C. sakazakii* and found that using this part of the sequence it was easier to isolate and distinguish *C. sakazakii* than with the 16S rRNA sequence. Therefore, the *gyrB* gene is a DNA target suitable for quantitative molecular detection of *C. sakazakii*.

Many stress factors may cause pathogenic bacteria to enter the VBNC state during the production, processing, and storage of milk and dairy products. Suliantari and Dewanti [40] found that *C. sakazakii* was induced to enter VBNC conditions (i.e., 1/10 tryptic soy agar and stainless-steel pipeline transportation), affecting the production and processing of milk powder. Jiang et al. [41] studied the survival rate of *C. sakazakii* under temperature conditions utilized for milk powder preparation. The results showed that some *C. sakazakii* strains could maintain certain activities under a preparation temperature of 65 °C and enter the VBNC state. Gunasekera et al. [42] observed cell subsets in the VBNC state in pasteurized fresh milk and further reported that although a large number of cells could not form colonies due to heat treatment, they still displayed measurable metabolic activities, including gene transcription and translation, even remaining pathogenic. Entering the VBNC state is a survival mechanism evolved by bacteria under environmental pressure. Since bacteria in the VBNC state are nonculturable, methods to detect and identify such bacteria have attracted extensive attention. Yang et al. [43] reported that *E. sakazakii* was isolated from Tetra Pak pure milk. It can be seen that there is still a risk of *E. sakazakii* contamination in liquid milk after pasteurization. Zheng et al. [44] found that the heat tolerance of each strain of *C. sakazakii* was different, which makes the time required for inactivation of *C. sakazakii* with a concentration of 10^7^ CFU/mL at 60 °C different. However, they did not test for a VBNC state. In this study, a PMA-QLAMP-based detection technology was used to quantitatively monitor *C.* *sakazakii* concentrations, indicating that pasteurization of 10^6^ CFU/mL *C. sakazakii* induced the largest proportion of *C.* *sakazakii* in the VBNC state.

At present, the commonly used methods for the detection of viable strains of *Cronobacteria sakazakii* are the culture method, and DNA binding dye and nucleic acid aptamer binding molecular detection methods, etc. Yuan et al. [45] established a biosensor based on RT-PCR for the electrochemical detection of viable *C. sakazakii* with a sensitivity of 7.68 × 10^3^ CFU/mL and at 2.4 × 10^7^ CFU/mL–3.84 × 10^4^ CFU/mL range for accurate quantification. Based on the aptamer ribozyme, a visual and rapid detection method for the viable strain of *C. sakazakii* was established with a sensitivity of 1.2 CFU/mL. Cao et al. [19] found that in the early or middle stage of milk fermentation, when inoculated with *C. sakazakii*, *C. sakazakii* could not be detected by the plate counting method in the later stage of fermentation but established that PMA-qPCR technology could detect the presence of viable *C. sakazakii*, indicating that *C. sakazakii* entered a VBNC state after milk fermentation. In this study, the detection limit of PMA-QLAMP for viable *C. sakazakii* was 4.1 × 10^2^ CFU/mL in pure culture and 4.3 × 10^2^ CFU/mL in the 10% PIF solution and the base material of *C. sakazakii* growth has almost no effect on the detection limit of PMA-QLAMP.

## 5. Conclusions

The PMA-QLAMP method established by the *gyrB* gene of *C. sakazakii* single-copy species-specific sequence design primers has strong specificity and high sensitivity. It can accurately quantify *C. sakazakii* viable bacteria with a range of 4.1 × 10^8^–4.1 × 10^2^ CFU/mL. The quantitative determination of viable *C. sakazakii,* including the VBNC state of *C. sakazakii*, obtains a more accurate detection result of viable bacteria than the traditional culture method of DFI plate counting. It is a specific, sensitive, accurate, and reliable detection method of *C. sakazakii* VBNC state in pasteurized raw milk, and provides an early warning of potential contamination of PIF.

## Figures and Tables

**Table 1:** PMA pretreatment conditions of the orthogonal test.

Contents	Factors		
	A	B	C
	PMA Working Concentration (μg/mL)	Time of Dark Reaction (min)	Time of Exposure (min)
1	3	3	3
2	5	5	5
3	10	10	10

**Table 2:** Results of L9 (3^4^) orthogonal experiment.

Test Number	Factors				
	A	B	C	Empty Column	Inhibition Rate (%)
	PMA Working Concentration (μg/mL)	Time of Dark Reaction (min)	Time of Exposure (min)		
1	1	1	1	3	99.18
2	1	2	2	1	99.41
3	1	3	3	3	98.75
4	2	1	2	2	99.25
5	2	2	3	1	98.73
6	2	3	1	2	99.32
7	3	1	3	2	99.69
8	3	2	1	3	99.98
9	3	3	2	1	99.07
K1	99.1143601	99.37584556	99.49332454	99.07	
K2	99.09920152	99.37205592	99.24320801	99.42132	
K3	99.58048636	99.0461465	99.05751543	99.30384	
R	0.481284841	0.329699064	0.435809108	0.352437	

Note: K1 was the average value of the inhibition rate corresponding to level 1 under 3 factors (A, B, and C); K2 was the average value of the inhibition rate corresponding to level 2 under 3 factors (A, B, and C); K3 was the average value of the inhibition rate corresponding to level 3 under 3 factors (A, B, and C). R was the maximum value minus the minimum value of the average inhibition rate (K1, K2, and K3) under each factor (A or B or C). Factor A: PMA working concentration; factor B: dark reaction time; factor C: exposure time.

**Table 3:** Formation of *C. sakazakii* VBNC state during pasteurization.

Serial Dilutions of Viable *C. sakazakii* before Pasteurization (CFU/mL)	LG CFU/mL			VBNC State of *C. sakazakii* Formation Ratio (%)
	QLAMP	Plate Count	PMA-QLAMP	
10^7^	7.64 ± 0.02	5.73 ± 0.04	6.28 ± 0.04	3.11
10^6^	6.67 ± 0.04	3.83 ± 0.03	5.86 ± 0.03	15.32
10^5^	5.72 ± 0.01	2.32 ± 0.04	4.80 ± 0.02	11.91
10^4^	4.8 ± 0.03	0	2.93 ± 0.01	1.38
10^3^	3.87 ± 0.02	0	1.08 ± 0.03	0.16
10^2^	2.8 ± 0.02	0	0	0
10^1^	1.76 ± 0.04	0	0	0

**Table 4:** PMA-QLAMP test results of viable *C. sakazakii* in raw milk before and after pasteurization.

Range of Ct Values (min)	Range of Viable Bacterial Concentration (CFU/mL)	Number of Positive Samples of Viable *C. sakazakii* in Raw Milk Detected by PMA-QLAMP		Positive Detection Rate of PMA-QLAMP (%)
		Before Pasteurization	After Pasteurization	
>24.24 and <28.12	<4.3 × 10^3^ and >4.3 × 10^2^	3	0	13.33
>28.12 and <34.28	<4.3 × 10^2^ and >4.3 × 10^1^	2	0	
>34.28 and <40	<4.3 × 10^1^ and >4.3 × 10^0^	3	0	

## Data Availability

The data presented in this study are available on request from the corresponding author.
